# Supplementary material for: Genetics behind Cerebral Disease with Ocular Comorbidity: Finding Parallels between the Brain and Eye Molecular Pathology
Source: Int J Mol Sci. 2022 Aug 26;23(17):9707. doi: 10.3390/ijms23179707 (PMC9456058; doi:10.3390/ijms23179707)
Supplement: Supplementary file 1 [file ijms-23-09707-s001.zip › Table S1.pdf]

Table S1. Pathomechanics of Joubert Syndrome

Subtypes [255][1]	Phenotype[1]	Phenotype OMIM[2] Number	Gene or Susceptibility Locus	Population of Founder Mutation	Chromosomal Location	Gene OMIM[2] Number	Protein	Molecular Level[3]	Affected Region or Effects on Brain[4]	Effects on Eye[5]	Inheritance Mode[6]	% of JBTS[1]	Reference
**Gene or Susceptibility Locus w/ Phenotype Number in OMIM Database**													
pure JBTS	JBTS8	612291	ARL13B	-	3q11.1-q11.2	608922	ADP Ribosylation Factor Like GTPase 13B	SHH signaling<br>GPCR signaling	MTS<br>cerebellar granule neurons<br>cerebral cortex	retinopathy	AR	<1	[89, 453, 454]
	JBTS13	614173	TECT1	-	12q24.11	609863	TECT1	TZ [88]	MTS<br>hypoplasia<br>gyration of the frontal lobes	rare association	AR	<1	[90, 455]
	JBTS15	614464	CEP41	-	7q32.2	610523	Centrosomal protein of 41 kDa	BB	MTS<br>ataxia<br>psychomotor delay<br>mental retardation	rare association	AR	<1	[91]
	JBTS22	615665	PDE6D	-	2q37.1	602676	Retinal rod rhodopsin-sensitive cGMP 3',5'-cyclic phosphodiester-ase subunit delta	ciliary trafficking	MTS<br>INPP5E prenyl-binding-dependent trafficking	rare association	AR	<1	[92, 456]
	JBTS25	616781	CEP104	-	1p36.32	616690	Centrosomal Protein Of 104 KDa	axoneme tip	MTS<br>delayed psychomotor development<br>oculomotor apraxia	rare association	AR	<1	[455]
	JBTS26	616784	KATNIP	-	16p12.1	616650	Katanin-Interacting Protein	BB	MTS<br>cerebellar hypoplasia<br>oculomotor apraxia	nystagmus<br>hypertelorism<br>cone dystrophy	AR	<1	[457, 458]
	JBTS27	617120	B9D1	-	17p11.2	614144	B9 Domain Containing 1	TZ	MTS<br>unspecified dysmorphic features<br>ataxia<br>oculomotor abnormalities	rare association	AR	<1	[95]
	JBTS30	617622	ARMC9	-	2q37	617612	LisH domain-containing protein ARMC9	BB	MTS<br>hypopituitarism<br>hypoplasia<br>bifid uvula<br>abnormal brainstem<br>dysplasia of the superior cerebellar folia	RD	AR	<1	[96]
	JBTS32	617757	SUFU	-	10q24	607035	Suppressor of fused homolog	ciliary tip	MTS<br>hypoplasia<br>elongated superior cerebellar peduncles<br>deepened interpeduncular fossa	rare association	AR	<1	[97]
	JBTS33	617767	PIBF1	-	13q21	607532	Progesterone-induced-blocking factor 1	BB-centrosome	MTS<br>hypoplasia<br>dysplasia of superior cerebellum<br>thickening of superior cerebellar peduncles<br>deep interpeduncular fossa	rare association	AR	<1	[459-461]

									bilateral polymicrogyria in the parietal and temporal areas<br>ventriculomegaly				
	JBTS35	618161	ARL3	-	10q24	604695	ADP-ribosylation factor-like protein 3	lipidated protein transport	MTS<br>hypoplasia<br>elongated superior cerebellar peduncles<br>oculomotor apraxia	night blindness<br>progressive visual impairment	AR	<1	[99]
	JBTS36	618763	FAM149B1	-	10q22	618763	Family with sequence similarity 149 member B1	ciliary tip	MTS<br>oculomotor apraxia	rare association	AR	<1	[100]
	JBTS37	619185	TOGARAM1	-	14q21	617618	TOGARAM1	longer cilia	MTS<br>hydrocephalus<br>cerebellar hypoplasia<br>lissencephaly	rare association	AR	<1	[101, 462]
	JBTS38	619476	KIAA0753	-	17p13	617112	KIAA0753	unknown	MTS<br>pituitary abnormalities	rare association	AR	<1	[102]
	JBTS39	619562	TMEM218	-	11q24.2	619285	Transmembrane Protein 218	ciliopathy	MTS<br>occipital encephalocele	rare association	AR	<1	[103]
	JBTS40	619582	IFT74	-	9p21.2	608040	Intraflagellar transport protein 74 homolog	IFT	MTS<br>oculomotor apraxia	rare association	AR	<1	[104]
**mixed JBTS w/retinal disease**	JBTS1	213300	INPP5E	-	9q34.3	613037	Phosphatidylinositol polyphosphate 5-phosphatase type IV	PDGFR-α signaling	MTS<br>cerebellar granule neurons<br>cerebral developmental defects	RD	AR	2-4	[105, 130, 463, 464]
	JBTS2	608091	TMEM216	Ashkenazi Jewish	11q12.2	613277	Transmembrane Protein 216	TZ	MTS<br>hypoplasia<br>cortical heterotopias	photoreceptor degeneration	AR	~2-3	[106, 130, 271, 464-466]
	JBTS3	608629	AHI1	-	6q23.3	608894	Jouberin	BB	MTS<br>cortical polymicrogyria	RD<br>retina degernation	AR	~7-10	[107, 130, 462, 467-470]
	JBTS4	609583	NPHP1	-	2q13	607100	Nephrocystin-1	TZ	MTS<br>hypo/aplasia	retinopathy	AR	~1-2	[108, 130, 464]
	JBTS5	610188	CEP290	Japaness	12q21.32	610142	Centrosomal Protein of 290kDa	TZ	MTS<br>cerebellar granule neurons	RP<br>retinal coloboma<br>vision reduction	AR	7-10	[109, 130, 471-475]
	JBTS7	611560	KIAA1005	-	16q12.2	610937	Retinitis pigmentosa GTPase regulator interacting protein 1-like	TZ	MTS<br>hypo/aplasia	RP	AR	1-4	[110, 130, 464, 476-478]
	JBTS9	612285	KIAA1345	-	4p15.32	612013	Coiled-Coil And C2 Domain Containing 2A	TZ	MTS<br>hypo/aplasai<br>mental retartation	RP	AR	~8-11%	[111, 130, 464, 479, 480]
	JBTS14	614424	TMEM237	Canadiun Hutterite	2q33.1	614423	Transmembrane Protein 237	TZ<br>WNT signaling	MTS<br>hypo/aplasai<br>encephalocele<br>hydrocephalus	optic disc anomaly<br>abnormal eye movement	AR	<1	[112, 481, 482]
	JBTS16	614465	TMEM138	-	11q12.2	614459	Transmembrane protein 138	TZ	MTS<br>telencephalon<br>rhombencephalon<br>cranial nerve ganglia	retinal dysplasia	AR	<1	[113]

	JBTS20	614970	TMEM231	French Cana-<br>dian	16q23.1	614949	Transmembrane Protein 231	TZ	MTS<br>breathing abnormalities<br>oculomotor apraxia	retinopathy	AR	<1	[114]
	JBTS28	617121	MKS1	-	17q2	609883	MKS Transition Zone Complex<br>Subunit 1	TZ	MTS<br>ataxia	RD	AR	~2-6	[95, 130, 464,<br>483]
	JBTS29	617562	TMEM107	-	17p13	616183	Transmembrane protein 107	TZ	MTS<br>oculomotor apraxia	retinopathy	AR	<1	[115]
**mixed JBTS w/o retinal disease**	JBTS6	610688	TMEM67	-	8q22.1	609884	Meckelin	TZ	MTS<br>hypo/aplasia<br>occipital encephalocele	rare association	AR	~6-20	[116, 130,<br>464, 480, 484-<br>487]
	JBTS10	300804	OFD1	-	Xp22.2	611951	Oral-Facial-Digital Syndrome 1<br>Protein	SHH signaling	MTS<br>embryonic neural tube patterning	rare association	XLR	<1	[117, 488]
	JBTS12	200990	KIF7	-	15q26.1	611254	Kinesin-like protein KIF7	SHH signaling	MTS<br>fetal hydrolethalus<br>acrocallosal syndromes<br>macrocephaly	delayed photorecptor outer segment morphogenesis<br>ocular coloboma	AR	<1	[118, 489-<br>492]
	JBTS17	614615	C5ORF42	French-Cana-<br>dian<br>Dutch	5p13.2	614571	Ciliogenesis and planar polarity<br>effector 1	SHH signaling	MTS<br>cerebellum developmental defects<br>macrocephaly	rare association	AR	8-14	[16, 119, 130,<br>464, 493-495]
	JBTS18	614815	TCTN3	-	10q24.1	613847	Tectonic 3	TZ<br>SHH signaling	MTS<br>nerual tube patterning	rare association	AR	<1	[120, 496]
	JBTS19	614844	NPHP14	-	16q12.1	604557	Zinc finger protein 423	DDR signaling	MTS<br>hypo/aplasia<br>purkinje cell progenitor development<br>hindbrain chorid plexus development	rare association	AR<br>AD	<1	[121, 497,<br>498]
	JBTS21	615636	CSPP1	Hutterite	8q13.1-q13.2	611654	Centrosome/spindle pole-associ-<br>ated protein 1	TZ<br>SHH signaling	MTS<br>abnormal mid-hid brain development	rare association	AR	2-4	[122-124, 130,<br>464]
	JBTS23	616490	KIAA0586	-	14q23.1	610178	Protein TALPID3	BB<br>SHH signaling	MTS<br>neuronal apoptosis<br>neural tube defects<br>neural tube patterning	ophthalmological defects	AR	~2-7	[125-127, 464,<br>499, 500]
	JBTS24	616654	TCTN2	-	12q24.31	613846	Tectonic 2	TZ	MTS<br>hypo/aplasia<br>delayed psychomotor development<br>polymicrogyria<br>pachygyria<br>myelination defects<br>encephalocele	nystagmus<br>hyperopia	AR	~1	[128, 501]
	JBTS31	617761	CEP120	-	5q23	613446	Centrosomal protein of 120 kDa	BB<br>SHH signaling	MTS<br>ataxia	rare association	AR	<1	[129, 502]
	JBTS34	614175	B9D2	-	19q13	611951	B9 domain-containing protein 2	TZ	MTS<br>occipital encephalocele	poor pupillary response to light	AR	<1	[130]
**Gene or Susceptibility Locus w/o Phenotype Number in OMIM Database**													

NA	NA	TCTN1	-	12q24.11	NA	Tectonic 1	TZ<br>SHH signaling	MTS	unknown	unknown	<1	[90]
NA	NA	TTC21B/NPHP12	-	2q24.3	NA	Tetratricopeptide Repeat Domain 21B	IFT-A complex<br>SHH signaling	MTS	unknown	unknown	<1	[131, 503]
NA	NA	C2CD3	-	11q13.4	NA	C2 domain-containing protein 3	unknown	MTS	unknown	unknown	<1	[130]
NA	NA	HYLS1	-	11q24.2	NA	Hydrolethalus syndrome protein 1	unknown	MTS	unknown	unknown	<1	[132]
NA	NA	IFT80	-	3q25.33	NA	Intraflagellar transport protein 80 homolog	unknown	MTS	unknown	unknown	<1	[132]
NA	NA	CELSR2	-	1p13.3	NA	Cadherin EGF LAG seven-pass G-type receptor 2	unknown	MTS	unknown	unknown	<1	[132]
NA	NA	POC1B	-	12q21.33	NA	POC1 centriolar protein homolog B	unknown	MTS	unknown	unknown	<1	[502]
NA	NA	IFT172	-	2p23.3	NA	Intraflagellar transport protein 172 homolog	unknown	MTS	unknown	unknown	<1	[130]

[1] JBTS indicates Joubert syndrome.

[2] OMIM indicates Online Mendelian Inheritance in Man; and NA, not available in the reference.

[3] SHH indicates Sonic Hedgehog; WNT, Wingless and Int-1; GPCR, G protein-coupled receptor; DDR, DNA-damage response; BB, basal body; IFT, intraflagellar transport; and TZ, transition zone.

[4] MTS indicates molar tooth sign.

[5] RD indicates retinal dystrophy; and RP, retinitis pigmentosa.

[6] AD indicates autosomal dominant; AR, autosomal recessive; and XLR, X-linked recessive.
