# Supplementary material for: Genetics behind Cerebral Disease with Ocular Comorbidity: Finding Parallels between the Brain and Eye Molecular Pathology
Source: Int J Mol Sci. 2022 Aug 26;23(17):9707. doi: 10.3390/ijms23179707 (PMC9456058; doi:10.3390/ijms23179707)
Supplement: Supplementary file 1 [file ijms-23-09707-s001.zip › Table S2.pdf]

Table S2. Pathomechanics of Bardet Biedl Syndrome

Phenotype[1]	Phenotype OMIM[2] Number	Gene or Susceptibility Locus	Chromosomal Location	Gene OMIM[2] Number	Protein	Molecular Level[3]	Affected Region or Effects on Brain	Effects on Eye[4]	Inheritance Mode[5]	% of BBS[1] [22]	Reference
**BBS w/ RD and Cognitive Impairment**											
BBS1	209900	BBS1	11q13.2	209901	Bardet-Biedl syndrome 1 protein	BBSome protein	cognitve impairment<br>speech disorder,<br>speech delay<br>developmental delay<br>mental retardation<br>ataxia<br>poor coordination	RD<br>RP<br>retinal degeneration<br>strabismus<br>cataracts	AR<br>DR	23.0	[133]
BBS2	615981	BBS2	16q13	606151	Bardet-Biedl syndrome 2 protein	BBSome protein	cognitive impairment	RD<br>retinal degeneration	AR	8.0	[133, 505]
BBS3	600151	ARL6	3q11.2	608845	ADP-ribosylation factor-like protein 6	GTPase	cognitive impairment<br>sdlayed development<br>mental retardation	RD<br>RP<br>night blindness<br>severe impairment of vision	AR	0.4	[133, 506]
BBS5	615983	BBS5	2q31.1	603650	Bardet-Biedl syndrome 5 protein	BBSome protein	cognitive impairment	RD<br>retinal macular changes	AR	0.4	[133, 507, 508]
BBS10	615987	BBS10	12q21.2	610148	Bardet-Biedl syndrome 10 protein	part of chaperonin complex	cognitive impairment	RD	AR	20.0	[133, 509]
BBS11	615988	TRIM32	9q33.1	602290	E3 ubiquitin-protein ligase TRIM32	E3 ubiquitin ligase	cognitive impairment	RD<br>retinopathy	AR	0.1	[133, 510]
BBS12	615989	BBS12	4q27	610683	Bardet-Biedl syndrome 12 protein	part of chaperonin complex	cognitive impairment	RD<br>cataracts	AR	1.0	[19, 133]
BBS16	615993	SDCCAG8	1q43-q44	613524	Serologically defined colon cancer antigen 8	BB	cognitive impairment	RD<br>RP<br>retinal degeneration<br>affected rod function	AR	<0.1	[133, 511, 512]
BBS17	615994	LZTFL1	3p21.31	606568	Leucine zipper transcription factor-like protein 1	BBSome-interacting protein	cognitive impairment	RD<br>RP	AR	<0.1	[133, 513, 514]
BBS18	615995	BBIP1	10q25.2	613605	BBSome-interacting protein 1	BBSome protein	cognitive impairment	RD<br>RP<br>cataracts<br>severe visual impairment	AR	<0.1	[133, 513, 515]
BBS19	615996	IFT27	22q12.3	615870	Intraflagellar transport protein 27 homolog	part of IFT-B complex	cognitive impairment	RD<br>RP<br>strabismus	AR	<0.1	[133, 513, 516, 517]
BBS21	617406	CFAP418	8q22.1	614477	Cilia- and flagella-associated protein 418	cilia	cognitive impairment	RD<br>RP<br>myopic degenerative changes<br>myopia<br>minimal photopic response	AR	<0.1	[133, 518, 519]

								moderately delayed or depressed scotopic response<br>macular retinal thinning			
BBS22	617119	IFT74	9p21.2	608040	Intraflagellar transport protein 74 homolog	part of IFT-B complex	cognitive impairment	RD<br>RP<br>macular hypopigmentation<br>granular appearance<br>reduced central vision<br>night blindness	AR	<0.1	[134, 520-522]
**BBS w/ RD but w/o Cognitive Impairment**											
BBS4	615982	BBS4	15q24.1	600374	Bardet-Biedl syndrome 4 protein	BBSome protein	anosmia	RD<br>retinal degeneration<br>night blindness<br>reduced VA	AR	2.0	[133, 523, 524]
BBS6	605231	MKKS	20p12.2	604896	McKusick-Kaufman/Bardet-Biedl syndromes putative chaperonin	part of chaperonin complex	mental retardation	RD<br>RP<br>reduced VA<br>photophobia	AR	6.0	[133, 507, 525]
BBS7	615984	BBS7	4q27	607590	Bardet-Biedl syndrome 7 protein	BBSome protein	mental retardation	RD<br>RP	AR	2.0	[133, 526]
BBS8	615985	TTC8	14q31.3	608132	Tetratricopeptide repeat protein 8	BBSome protein	speech impediment<br>developmental delay	RD<br>RP	AR	1.0	[133, 527]
BBS9	615986	PTHB1	7p14.3	607968	Protein PTHB1	BBSome protein	mental retardation	RD<br>RP<br>retinopathy	AR	6.0	[133, 528]
BBS13	615990	MKS1	17q22	609883	Meckel syndrome type 1 protein	centriole migration	delayed development<br>mental retardation	RD<br>RP<br>retinal degeneration	AR	4.5	[133, 529]
BBS14	615991	CEP290	12q21.32	610142	Centrosomal protein of 290 kDa	BB	mental retardation	RD<br>RP<br>retinal degeneration	AR	1.0	[133, 530]
BBS20	619471	IFT172	2p23.3	607386	Intraflagellar transport protein 172 homolog	part of IFT-B complex	intracranial hypertension	RD<br>RP<br>strabismus<br>hyperopia<br>astigmatism<br>night blindness	AR	<0.1	[133, 531-533]
BBS w/o RD and Cognitive Impairment											
BBS15	615992	WDPCP	2p15	613580	WD repeat-containing and planar cell polarity effector protein fritz homolog	BB	unknown	unknown	AR	1.0	[133]

[1] BBS indicates Bardet Biedl syndrome.
[2] OMIM indicates Online Mendelian Inheritance in Man.

[3] BB indicates basal body; and IFT, intraflagellar transport.

[4] RD indicates retinal dystrophy; RP, retinitis pigmentosa; and VA, visual acuity.

[5] AR indicates autosomal recessive; and DR, digenic recessive.
