# Supplementary material for: Genetics behind Cerebral Disease with Ocular Comorbidity: Finding Parallels between the Brain and Eye Molecular Pathology
Source: Int J Mol Sci. 2022 Aug 26;23(17):9707. doi: 10.3390/ijms23179707 (PMC9456058; doi:10.3390/ijms23179707)
Supplement: Supplementary file 1 [file ijms-23-09707-s001.zip › Table S3.pdf]

Table S3. Pathomechanics of Neuromyelitis Optica Spectrum Disorder

Location of Protein	Gene or Susceptibility Locus	Chromosomal Location	SNP or Allele	Protein	Molecular Level	Only Found in AQP4-IgG+	Reference
Nucleus	EHMT2	6p21.33		EHMT2	H3K9		[150]
	MSH5	6p21.33		MutS protein homolog 5	DNA mismatch repair and meiotic recombination		[150]
	NOP16	5q35.2		Nucleolar protein 16	downregulation of ribosome functions	V	[151]
Cytoplasm	PRF1	10q22.1		perforin	cytotoxicity of NK		[152]
	ATG5	6q21	rs548234	Autophagy protein 5	autophagy		[153]
	CYP27B1	12q14.1	rs703842<br>rs10876994	25-hydroxyvitamin D-1 alpha hydroxylase	vitamin D metabolism		[538]
	CYP7A1	8q12.1	rs1457043<br>rs3808607<br>rs3824260	Cytochrome P450 7A1	transcriptional activity		[154, 155]
Surface	NECL2	11q23.3	rs770344177	NECL2	signaling in T cells and NK		[156]
	AQP4	18q11.2	rs151244<br>rs335929<br>rs1058424	Aquaporin-4	complement-dependent cytotoxicity	V	[157, 452]
	FCRL3	1q23.1	rs945635<br>rs3761959<br>rs2282284<br>rs7528684	Fc receptor-like protein 3	immunoreceptor tyrosine-based inhibitory motif<br>immunoreceptor tyrosine-based activation motif		[158]
	GPC5	13q31.3	rs1411751<br>rs9523762	Glypican-5	cell to cell signaling	V	[159]
	KCNMA1	10q22.3	rs1516512	Calcium-activated potassium channel subunit alpha-1	BK channels		[160]
	SLC29A1	6p21.1	rs507964	Equilibrative nucleoside transporter 1	erythrocyte concentration of 6-methylmercaptopurine nucleotides		[161]
	SLC28A3	9q21.33	rs10868138<br>rs12378361	Solute carrier family 28 member 3	erythrocyte concentration of 6-thioguanine nucleotides		[161]
	SLC44A4	6p21.33		TPPT	the uptake of choline by cholinergic neurons		[150]
	CD25	10p15.1	rs2104286	Interleukin-2 receptor subunit alpha	differentiate into antibody-secreting cells		[162, 535]
	CD58	1p13.1	rs1016140<br>rs1335532<br>rs2300747<br>rs6677309<br>rs12044852<br>rs12288280<br>rs56302466	Lymphocyte function-associated antigen 3	T-cell hyperactivity	V	[160, 163, 536-538]
	CD127	5p13.2	rs6897932	Interleukin-7 receptor subunit alpha	T cell maturation and survival	V	[166, 538]
	CD226	18q22.2	rs763361	CD226 antigen	regulatory T cells		[165, 167]

	HLA-A	6p22.1	*01	HLA class I histocompatibility antigen, A alpha chain	CD8+ cytotoxic T-cell response	V	[168, 539]
	HLA-B	6p21.33	*08	HLA class I histocompatibility antigen, B alpha chain	CD8+ cytotoxic T-cell response	V	[539, 540]
	HLA-C	6p21.33	*15:02	HLA class I histocompatibility antigen, C alpha chain	CD8+ cytotoxic T-cell response		[169, 539]
	HLA-DPB1	6p21.32	*05:01	HLA class II histocompatibility antigen, DP beta 1 chain	transcription levels of HLA-DP gene		[81, 541]
	HLA-DRB1	6p21.32	*01:02<br>*02:01<br>*03<br>*04:05<br>*08:02<br>*10<br>*14:06<br>*16:02	HLA class II histocompatibility antigen, DRB1 beta chain	regulating B cell		[168-172]
	HLA-DQA1	6p21.32	*01:02<br>*01:05<br>*05:01<br>*05:03	HLA class II histocompatibility antigen, DQ alpha 1 chain	regulating B cell		[168, 170, 173, 174]
	HLA-DQB	6p21.32	*35:14<br>*39:06	HLA class II histocompatibility antigen, DQ beta 1 chain	regulating B cell		[168, 172]
	HLA-DQB1	6p21.32	*02<br>*04:02	HLA class II histocompatibility antigen, DQ beta 1 chain	regulating B cell	V	[168, 172, 175]
**Secreted**	CFB	6p21.33		Complement factor B	alternative pathway of complement activation	V	[150]
	C4B	6p21.3		Complement C4-B	lower C4	V	[176, 542]
